# Effects of matrix support implementation on the community mental health network in a medium-sized Brazilian municipality: a repeated cross-sectional study

**DOI:** 10.1590/0102-311XEN038925

**Published:** 2025-08-22

**Authors:** Carlos Alberto dos Santos Treichel, Maria Giovana Borges Saidel, Ana Laura Salomé Lourencetti, Sulamita Gonzaga Silva Amorim, Lívia Penteado Pinheiro, Rosana Teresa Onocko-Campos

**Affiliations:** 1 Escola de Enfermagem, Universidade de São Paulo, São Paulo, Brasil.; 2 Faculdade de Enfermagem, Universidade Estadual de Campinas, Campinas, Brasil.; 3 Faculdade de Ciências Médicas, Universidade Estadual de Campinas, Campinas, Brasil.

**Keywords:** Mental Health, Community Mental Health Services, Primary Care, Saúde Mental, Serviços Comunitários de Saúde Mental, Atenção Primária, Salud Mental, Servicios Comunitarios de Salud Mental, Atención Primaria

## Abstract

Given the centrality of comprehensive care and network organization as key outcomes of matrix support, this study aims to examine changes in the operation of the community mental health network in Itatiba, São Paulo State, Brazil, after the implementation of matrix support, focusing on three main components: (i) users’ profile; (ii) identification of available therapeutic interventions; and (iii) analysis of the clinical care received by participants across the studied services. This is a repeated cross-sectional, document-based descriptive study with two measurement points (T0 and T1). Data for T0 were collected between May and July 2019, and for T1 between November and December 2021. A total of 1,958 records were analyzed at T0 and 1,431 at T1, using descriptive statistical analysis to estimate absolute and relative frequencies for each variable. The findings indicate substantial changes in referral patterns following matrix support implementation, with a decrease in spontaneous demand and a greater role of primary care in coordinating access to specialized mental health services. Improvements were also observed in clinical care, with increased attention to vital signs measurement, anamnesis, physical exams, and documentation of smoking, alcohol, and psychoactive substance use. These results suggest that matrix support contributed to a more structured and effective mental health network, enhancing integration between care levels and promoting more comprehensive and accessible care.

## Resumo

Dada a centralidade do cuidado integral e da organização da rede como desfechos chave do apoio matricial, este estudo analisa as mudanças no funcionamento da rede comunitária de saúde mental em Itatiba, São Paulo, Brasil, após a implementação do apoio matricial, com foco em três componentes principais: (i) perfil dos usuários; (ii) identificação das intervenções terapêuticas disponíveis; e (iii) análise do atendimento clínico recebido pelos participantes nos serviços estudados. Trata-se de um estudo descritivo transversal repetido, documental, com dois pontos de medida (T0 e T1). Os dados para T0 foram coletados entre maio e julho de 2019 e para T1 entre novembro e dezembro de 2021. Foram analisados 1.958 registros em T0 e 1.431 em T1, utilizando-se análise estatística descritiva para estimar frequências absolutas e relativas para cada variável. Os achados indicam mudanças substanciais nos padrões de encaminhamento após a implementação do apoio matricial, com diminuição da demanda espontânea e maior protagonismo da atenção básica na coordenação do acesso aos serviços especializados de saúde mental. Também foram observadas melhorias no atendimento clínico, com maior atenção à mensuração dos sinais vitais, anamnese, exames físicos e documentação do tabagismo, álcool e uso de substâncias psicoativas. Esses resultados sugerem que o apoio matricial contribuiu para uma rede de saúde mental mais estruturada e efetiva, favorecendo a integração entre os níveis de atenção e promovendo um cuidado mais integral e acessível.

## Resumen

Dada la importancia de la atención integral y la organización en red como resultados clave del apoyo matricial, este estudio tiene como objetivo examinar los cambios en el funcionamiento de la red de salud mental comunitaria en Itatiba, São Paulo, Brasil, tras la implementación del apoyo matricial, centrándose en tres componentes principales: (i) el perfil de los usuarios; (ii) la identificación de las intervenciones terapéuticas disponibles; y (iii) el análisis de la atención clínica recibida por los participantes en los servicios estudiados. Se trata de un estudio descriptivo transversal repetido, basado en documentos, con dos puntos de medición (T0 y T1). Los datos para T0 se recopilaron entre mayo y julio de 2019, y los de T1 entre noviembre y diciembre de 2021. Se analizaron un total de 1.958 registros en T0 y 1.431 en T1, utilizando análisis estadísticos descriptivos para estimar las frecuencias absolutas y relativas de cada variable. Los hallazgos indican cambios sustanciales en los patrones de derivación tras la implementación del apoyo matricial, con una disminución de la demanda espontánea y un mayor papel de la atención primaria en la coordinación del acceso a los servicios especializados de salud mental. También se observaron mejoras en la atención clínica, con un aumento en la toma de signos vitales, la anamnesis, los exámenes físicos y la documentación del consumo de tabaco, alcohol y sustancias psicoactivas. Estos resultados sugieren que el apoyo matricial contribuyó a una red de salud mental más estructurada y eficaz, mejorando la integración entre los niveles de atención y promoviendo una atención más integral y accesible.

## Introduction

Inadequate care for individuals with mental health disorders significantly contributes to the mortality disparity within this population, which the World Health Organization (WHO) has identified as one of the major global health challenges of the millennium ^1^. Estimates indicate that people living with mental disorders experience mortality rates twice to thrice higher than the general population and have a reduced life expectancy of 10 to 30 years ^2^ ^,^ ^3^ ^,^ ^4^.

Among the factors contributing to this scenario are the weak integration of healthcare networks and the shortage of adequately trained professionals to provide appropriate care in primary care settings ^5^ ^,^ ^6^. Consequently, the capacity to identify and manage cases at the primary care remains limited, overloading specialized services and, consequently, delaying access to mental health care ^7^ ^,^ ^8^ ^,^ ^9^ ^,^ ^10^.

To address these challenges, several countries have implemented network integration mechanisms intending to foster collaboration between primary care professionals and specialists ^11^. Notable initiatives such as shared care and collaborative care models have been successfully adopted in Australia and Canada ^12^ ^,^ ^13^.

Shared and collaborative care are characterized by the joint participation of primary care professionals and specialists in patient management, facilitated by organizational arrangements. These models typically include the following components: (i) the designation of case coordination responsibilities to professionals within the primary care team - such as physicians or nurses - who conduct systematic and structured clinical and care management interventions; (ii) the implementation of mechanisms that facilitate regular communication and collaboration between primary care providers and mental health specialists; and (iii) the development of strategies for collecting and sharing information about patient progress between different levels of care, especially between primary care and specialized mental health services ^12^ ^,^ ^13^.

In Brazil, to enhance comprehensive care and foster integrative collaboration across specialties and professions, national health networks have incorporated the matrix support model since 2008. As proposed by Campos & Domitti ^14^, matrix support represents an innovative pedagogical-therapeutic intervention designed to promote new relational patterns among healthcare professionals, facilitate information exchange, and increase co-responsibility in patient care. Furthermore, matrix support teams serve as specialized backup units, reducing unnecessary referrals to higher levels of care and enhancing the problem-solving capacity of primary care teams ^14^.

Recognizing the potential of matrix support to improve the quality of mental health services, the municipality of Itatiba, São Paulo, adopted this approach with the primary objectives of reorganizing care pathways and strengthening the local network structure in line with psychosocial care guidelines. This initiative sought to create a more integrated and efficient system, improving coordination between primary care and specialized mental health services to ensure a more comprehensive and effective response to the population’s needs.

Implementation of the matrix support in Itatiba took place between 2019 and 2021, guided by a Research Steering Committee (RSC). This deliberative body consisted of seven researchers affiliated with an academic institution, who synthesized and translated evidence to support implementation; five managers and mental health workers from municipal services; and two municipal administration representatives directly involved in coordinating mental health services. Together, they formed the matrix support implementation team ^15^.

Matrix support was implemented by a team of professionals linked to two Centers for Psychosocial Care (CAPS, acronym in Portuguese) in the municipality - one type II CAPS and the other a CAPS specialized in alcohol and other drugs (CAPS AD). This matrix team consists of psychiatrists, psychologists, occupational therapists, social workers, and works in an itinerant manner with primary care teams operating under the Family Health Strategy (FHS) model. Matrix support meetings occur monthly, involving two FHS teams at a time, with one of the meetings including three teams. Primary care physicians, nurses, and community health agents participate in these sessions, which last approximately two hours. During the meetings, complex cases undertaken by primary health teams are discussed to enhance the capacity of primary care to manage such cases with clinical and pedagogical support. The approach promotes shared responsibility between the teams and when the primary care unit’ resources are deemed insufficient, the case may be referred to specialized services. Nevertheless, the case remains under the joint responsibility of primary care via counter-referral mechanisms, thus ensuring longitudinal and integrated care.

Prior to implementation, a situational analysis of the municipal community mental health network (RAPS, acronym in Portuguese) was conducted. This assessment enabled the RSC to collaboratively develop a theory of change (ToC) to guide the implementation process. The ToC framework established intermediate and long-term outcomes, their causal relations, associated indicators, underlying assumptions, and the necessary implementation strategies to achieve them. Among the agreed-upon indicators, in addition to qualitative measures related to implementing and institutionalizing the matrix support, six quantitative indicators were defined to assess the quality and comprehensiveness of care and changes in network flow. These were considered key markers of matrix support effectiveness and shared care within the network ^16^.

Given the centrality of comprehensive care and network organization as key outcomes of matrix support, this study examines changes in the RAPS operation in Itatiba by focusing on three main components: (i) users’ profile; (ii) identification of available therapeutic interventions; and (iii) analysis of the clinical care received by participants across the studied services.

## Methods

### Study design

This is a repeated cross-sectional, document-based descriptive study with two measurement points (T0 and T1). Data for T0 were collected between May and July 2019, and for T1 between November and December 2021.

### Study population and participant recruitment

This study was conducted in Itatiba, a medium-sized municipality in São Paulo State with approximately 120,000 inhabitants, located 80Km from the state capital. In addition to hospital and emergency services, the municipality’s community network includes 19 primary care services, 13 of which operate under the FHS model and six under the traditional primary care model, and three specialized mental health services: one CAPS II, one CAPS AD, and an outpatient clinic.

All individuals affiliated with CAPS II, CAPS AD, and the outpatient clinic were deemed eligible. Inclusion criteria at T0 required at least one recorded visit to the services between May 2018 and May 2019, and at T1, at least one recorded visit between November 2020 and November 2021. Importantly, individuals at T0 and T1 could be the same, meaning that some patients may be included in both time points. Exclusion criterion was being a minor. Thus, the study included 1,958 records at T0 and 1,431 records at T1.

### Logistics

Data collection were conducted at the studied services between May and July 2019 for T0 and between November and December 2021 for T1. Data collection involved 16 previously trained collectors who study Psychology and Medicine, supervised by a nurse and a social worker. When inclusion criteria were met, collectors completed a specifically designed form based on available information in the medical records.

### Variables

To address the study objectives, the following variables were selected based on the medical records: sex (female, male); age in years (18-30, 31-45, 46-60, 61 or older); schooling level in years (0-4, 5-8, 9 or more, no record); paid employment (yes, no, no record); duration of care in months (1-6, 7-12, 13-36, 37-60, 61 or more); referral sources (spontaneous demand, primary care, hospital or emergency service, other specialized service, private services); mental health diagnosis (anxiety disorders, unipolar affective disorders, bipolar affective disorders, psychotic disorders, substance use disorders, intellectual disability and organic disorders, no diagnosis); number of mental health visits (1-3, 4-6, 6-12, 13 or more); therapeutic offerings (mental health medical consultation, individual care with a multidisciplinary team professional (MTP), group care, therapeutic/workshop activities, community activities, home care, physical activity); clinical care received (weight measurement, vital signs measurement, anamnesis and physical examination, laboratory tests, clinical treatments/referrals); hypertension (yes, no, no record); diabetes (yes, no, no record); smoking (yes, no, no record); alcohol use (yes, no, no record); use of other psychoactive substances (yes, no, no record).

The variables related to therapeutic offerings and clinical care received were evaluated based on records from the years preceding data collection, i.e., from May 2018 to May 2019 for T0 and from November 2020 to November 2021 for T1.

### Statistical analysis

Statistical analyses were performed using Stata 18 (https://www.stata.com). Descriptive statistical analysis was conducted with estimates of absolute and relative frequencies for each of the studied variables. As the lack of piece of information was considered relevant for discussion, missing data were computed and reported.

### Ethical procedures

This study was submitted to and approved by the Research Ethics Committee of the College of Medical Sciences at the State University of Campinas (opinion n. 3,793,771). It was conducted in accordance with Brazilian regulations and guidelines for research involving human subjects (*Resolution n. 466/2012* of the Brazilian National Health Council), as well as the provisions of the *Declaration of Helsinki*. Due to its document-based nature, the requirement for informed consent was formally waived by the ethics committee.

## Results

The study included 1,958 records at T0, of which 21.1% (n = 413) were linked to CAPS II, 18.3% (n = 359) to CAPS AD, and 52.7% (n = 1,032) to the outpatient clinic. At T1, there were 1,431 records, with 28.5% (n = 408) linked to CAPS II, 35.4% (n = 507) to CAPS AD, and 36.1% (n = 516) to the outpatient clinic. At both time points female users predominated at CAPS II (T0: 55%, n = 227; T1: 58.3%, n = 238) and at the outpatient clinic (T0: 71.8%, n = 741; T1: 70.7%, n = 365), whereas male patients predominated at CAPS AD (T0: 77.7%, n = 279; T1: 83.4%, n = 423). Table 1 presents a detailed characterization of the study participants regarding their sociodemographic characteristics, mental health diagnoses, referral sources, and service follow-up.

**Table 1:** Patient profile by sociodemographic, diagnosis, referral source, and follow-up characteristics at different time points (T0: n = 1,958; T1: n = 1,431).

Characteristics	T0			T1		
	CAPS II	CAPS AD	Outpatient clinic	CAPS II	CAPS AD	Outpatient clinic
	% (n)	% (n)	% (n)	% (n)	% (n)	% (n)
Sex						
Female	55.0 (227)	22.3 (80)	71.8 (741)	58.3 (238)	16.6 (84)	70.7 (365)
Male	45.0 (186)	77.7 (279)	28.2 (291)	41.7 (170)	83.4 (423)	29.3 (151)
Age (years)						
18-30	13.1 (54)	28.1 (101)	12.3 (127)	19.8 (81)	21.3 (108)	20.3 (105)
31-45	30.8 (127)	38.5 (138)	29.7 (306)	33.6 (137)	48.5 (246)	22.5 (116)
46-60	39.2 (162)	28.4 (102)	31.5 (325)	34.1 (140)	24.1 (122)	29.8 (154)
61 or older	16.9 (70)	5.0 (18)	26.5 (274)	12.2 (50)	6.1 (31)	27.3 (141)
Schooling (years)						
0-4	40.7 (168)	8.1 (29)	29.0 (299)	35.1 (143)	16.0 (81)	28.7 (148)
5-8	10.4 (43)	4.5 (16)	15.8 (163)	13.7 (56)	11.0 (56)	13.0 (67)
9 or more	25.2 (104)	4.7 (17)	25.1 (259)	42.6 (174)	20.7 (105)	19.4 (152)
No record	23.7 (98)	82.7 (297)	30.1 (311)	8.6 (35)	52.3 (265)	28.9 (149)
Paid work						
Yes	11.4 (47)	27.3 (98)	1.0 (10)	23.0 (94)	64.7 (328)	7.2 (37)
No	44.8 (185)	20.3 (73)	0.5 (5)	53.9 (220)	12.8 (65)	4.3 (22)
No record	43.8 (181)	52.4 (188)	98.5 (1,017)	23.0 (94)	22.5 (114)	88.6 (457)
Time in service (months)						
1-6	11.4 (47)	37.3 (134)	14.3 (148)	11.5 (47)	22.5 (114)	8.3 (43)
7-12	7.2 (30)	24.5 (88)	15.5 (160)	11.8 (48)	14.0 (71)	15.9 (82)
13-36	17.9 (74)	10.6 (38)	24.4 (252)	19.1 (78)	19.1 (97)	29.8 (154)
37-60	14.8 (61)	8.1 (29)	45.1 (465)	11.8 (48)	9.3 (47)	12.2 (63)
61 or more	48.7 (201)	19.5 (70)	0.7 (7)	45.8 (187)	35.1 (178)	33.7 (174)
Mental health diagnosis						
Anxiety disorders	16.0 (66)	12.5 (45)	55.5 (573)	23.3 (95)	16.2 (82)	42.2 (218)
Affective disorders (unipolar)	17.2 (71)	6.9 (25)	22.1 (228)	23.1 (94)	5.72 (29)	21.1 (109)
Affective disorders (bipolar)	17.7 (73)	2.2 (8)	4.0 (41)	32.6 (133)	6.5 (33)	8.7 (45)
Psychotic disorders	41.6 (172)	3.3 (12)	5.6 (58)	33.6 (137)	4.3 (22)	5.0 (26)
Substance abuse disorders	4.1 (17)	36.5 (131)	4.0 (41)	6.1 (25)	69.2 (351)	5.2 (27)
Mental retardation and organic disorders	13.1 (54)	3.1 (11)	6.2 (64)	11.5 (47)	2.9 (15)	10.3 (53)
No diagnosis	11.9 (49)	62.4 (224)	27.1 (280)	0.2 (1)	28.1 (142)	28.9 (149)
Referral source						
Spontaneous demand	15.5 (64)	50.4 (181)	71.7 (740)	9.8 (40)	56.2 (285)	14.7 (76)
Primary care	16.9 (70)	12.0 (43)	25.9 (267)	39.0 (159)	12.4 (63)	68.6 (354)
Hospital or emergency service	11.4 (47)	3.9 (14)	0.4 (4)	19.1 (78)	6.1 (31)	0.6 (3)
Other specialized service	49.9 (206)	8.9 (32)	1.0 (11)	27.9 (114)	20.3 (103)	15.3 (79)
Private services	6.3 (26)	24.8 (89)	1.0 (10)	4.2 (17)	4.9 (25)	0.8 (4)
Number of mental health visits received						
1-3	24.0 (99)	53.5 (192)	65.9 (680)	22.8 (93)	43.4 (220)	59.1 (305)
4-6	61.5 (254)	18.4 (66)	26.5 (274)	21.1 (86)	23.7 (120)	33.3 (172)
6-12	11.1 (46)	14.7 (53)	6.7 (69)	19.8 (81)	20.1 (102)	6.0 (31)
13 or more	3.4 (14)	13.4 (48)	0.9 (9)	36.3 (148)	12.8 (65)	1.6 (8)

CAPS AD: Center for Psychosocial Care specialized in alcohol and other drugs; CAPS II: Center for Psychosocial Care - type II.

No substantial changes were observed in the sociodemographic profile of the population served by the studied services between T0 and T1. However, it was noted a consistent lack of records related to education and paid employment which, although improved at CAPS II and CAPS AD, remained underreported at the outpatient clinic. On the other hand, the number of users attending the services for 61 months or more increased at CAPS AD (T0: 19.5%, n = 70; T1: 35.1%, n = 178) and the outpatient clinic (T0: 0.7%, n = 7; T1: 33.7%, n = 174).

Similar to the sociodemographic profile, we found no substantial changes regarding the mental health diagnostic profile of patients at each service. However, the proportion of undiagnosed patients decreased notably at CAPS II (T0: 11.9%, n = 49; T1: 0.2%, n = 1) and CAPS AD (T0: 62.4%, n = 224; T1: 28.1%, n = 142), with a significant increase in the diagnostic of substance abuse at CAPS AD (T0: 36.5%, n = 131; T1: 69.2%, n = 351).

Among the main changes observed, those related to the referral source of patients at each service stand out. Admissions due to spontaneous demand decreased at CAPS II (T0: 15.5%, n = 64; T1: 9.8%, n = 40) and the outpatient clinic (T0: 71.7%, n = 740; T1: 14.7%, n = 76), and the participation of primary care in referrals to these services increased significantly: from 16.9% (n = 70) to 39% (n = 159) at CAPS II and from 25.9% (n = 267) to 68.6% (n = 354) at the outpatient clinic. Interaction between specialized services also increased, with a decrease in patients referred from other specialized services at CAPS II (T0: 49.9%, n = 206; T1: 27.9%, n = 114) and an increase in such referrals at CAPS AD (T0: 8.9%, n = 32; T1: 20.3%, n = 103) and the outpatient clinic (T0: 1%, n = 11; T1: 15.3%, n = 79). Finally, the participation of private services in referring patients to CAPS AD decrease significantly, from 24.8% (n = 89) to 4.9% (n = 25).

Table 2 presents the therapeutic offerings and clinical care received by patients included in the study according to the point of care to which they were linked.

**Table 2:** Therapeutic interventions and clinical care received by patients, presented by point of care (T0: n = 1,958; T1: n = 1,431).

	T0			T1		
	CAPS II	CAPS AD	Outpatient clinic	CAPS II	CAPS AD	Outpatient clinic
	% (n)	% (n)	% (n)	% (n)	% (n)	% (n)
Therapeutic interventions						
Mental health medical consultation	99.3 (410)	58.2 (209)	97.0 (1,001)	97.1 (396)	81.5 (413)	98.6 (509)
Individual therapy sessions	90.1 (372)	89.9 (232)	28.4 (293)	89.2 (364)	97.8 (496)	72.1 (372)
Group therapy sessions	8.96 (37)	54.6 (196)	0.0 (0)	6.4 (26)	20.5 (104)	0.0 (0)
Therapeutic workshop/income generation activities	9.2 (38)	6.6 (24)	0.0 (0)	7.6 (31)	4.9 (25)	0.0 (0)
Community engagement activities	2.7 (11)	0.0 (0)	0.0 (0)	1.2 (5)	0.4 (2)	0.0 (0)
Home visits	6.3 (26)	0.0 (0)	0.0 (0)	12.0 (49)	7.9 (40)	0.0 (0)
Clinical care received						
Weight measurement	15.5 (64)	13.4 (48)	9.4 (97)	82.3 (336)	3.1 (16)	20.5 (106)
Vital signs measurement	7.3 (30)	39.3 (141)	4.2 (43)	81.9 (334)	5.9 (30)	36.1 (186)
Medical history and physical examination	59.1 (244)	61.8 (222)	14.4 (180)	73.3 (299)	13.2 (67)	80.6 (416)
Laboratory tests	16.2 (67)	12.5 (45)	12.2 (124)	14.9 (61)	13.8 (70)	39.1 (202)
Clinical treatments or referrals	17.4 (72)	8.1 (29)	8.5 (88)	28.4 (116)	15.2 (77)	40.5 (209)

CAPS AD: Center for Psychosocial Care specialized in alcohol and other drugs; CAPS II: Center for Psychosocial Care - type II.

Substantial changes were observed in the service delivery model at CAPS AD between T0 and T1. The proportion of patients receiving medical consultations with mental health professionals increased (T0: 58.2%, n = 209; T1: 81.5%, n = 413), followed by a rise in individual consultations provided by the multidisciplinary team (T0: 89.9%, n = 232; T1: 97.8%, n = 496). Conversely, the proportion of patients participating in group consultations decreased during the same period (T0: 54.6%, n = 196; T1: 20.5%, n = 104). Additionally, both CAPS AD and CAPS II showed a marked increase in the proportion of patients receiving home visits at T1 compared with T0 (CAPS AD - T0: 0%, n = 0; T1: 7.9%, n = 40; CAPS II - T0: 6.3%, n = 26; T1: 12%, n = 49), suggesting a shift towards more individualized and outreach-based care practices.

Among the changes in work processes, the increase in clinical care provided to patients at CAPS II and the outpatient clinic stands out, with significant improvements observed at the former. The proportion of patients who had their weight and vital signs measured at CAPS II increased from 15.5% (n = 64) to 82.3% (n = 336) and from 7.3% (n = 30) to 36.1% (n = 186), respectively. At the outpatient clinic, these proportions increased from 9.4% (n = 97) to 20.5% (n = 106) and from 4.2% (n = 43) to 36.1% (n = 186), respectively. Additionally, the proportion of patients undergoing anamnesis and physical examination at these services increased significantly; however, the same results were not observed for CAPS AD, in which most aspects of clinical care decreased.

Table 3 presents the proportions of recorded comorbidities, smoking, alcohol consumption, and other substance use among the patients included in the study according to the service point to which they were linked.

**Table 3:** Proportions of comorbidities, smoking, alcohol consumption, and other psychoactive substances among study patients, by point of care (T0: n = 1,958; T1: n = 1,431).

	T0			T1		
	CAPS II	CAPS AD	Outpatient clinic	CAPS II	CAPS AD	Outpatient clinic
	% (n)	% (n)	% (n)	% (n)	% (n)	% (n)
Hypertension						
Yes	17.7 (73)	4.7 (17)	5.8 (60)	18.1 (74)	4.9 (25)	9.1 (47)
No	42.4 (175)	27.8 (100)	2.2 (23)	47.6 (194)	19.5 (99)	9.3 (17)
No record	39.9 (165)	67.4 (242)	92.0 (949)	34.3 (140)	75.6 (383)	87.6 (452)
Diabetes						
Yes	12.3 (51)	1.9 (7)	2.7 (28)	13.3 (42)	2.0 (10)	5.8 (30)
No	45.3 (187)	29.0 (104)	3.1 (32)	52.9 (216)	19.7 (100)	4.7 (24)
No record	42.4 (175)	69.1 (248)	94.2 (972)	36.8 (150)	78.3 (397)	89.5 (462)
Smoking						
Yes	24.9 (103)	17.5 (63)	1.1 (11)	23.8 (97)	40.0 (203)	2.5 (13)
No	55.2 (228)	11.4 (41)	2.2 (23)	52.4 (214)	6.3 (32)	2.9 (15)
No record	19.9 (82)	71.1 (255)	96.7 (998)	23.8 (97)	53.7 (272)	94.6 (488)
Alcoholism						
Yes	13.1 (54)	61.0 (219)	0.5 (5)	6.6 (27)	84.4 (428)	3.9 (20)
No	65.6 (271)	4.2 (15)	2.0 (21)	70.6 (288)	5.9 (30)	3.9 (20)
No record	21.3 (88)	34.8 (125)	97.5 (1,006)	22.8 (93)	9.7 (49)	92.2 (476)
Other psychoactive substances						
Yes	8.2 (34)	56.3 (202)	0.5 (5)	12.0 (49)	74.6 (378)	13.9 (72)
No	67.3 (278)	6.9 (25)	0.1 (1)	63.7 (260)	11.6 (59)	0.8 (4)
No record	24.5 (101)	36.8 (132)	99.4 (1,026)	24.3 (99)	13.8 (70)	85.3 (440)

CAPS AD: Center for Psychosocial Care specialized in alcohol and other drugs; CAPS II: Center for Psychosocial Care - type II.

There were slight changes in the recording of comorbidities such as hypertension and diabetes. These changes were positive for CAPS II and the outpatient clinic, in which the proportion of patients without records for these conditions decreased, but negative for CAPS AD, which had a lower proportion of records in T1 compared with T0. On the other hand, record-keeping significantly improved at CAPS AD, showing a decrease in the proportion of patients without records for smoking (T0: 71.1%, n = 255; T1: 53.7%, n = 272), alcohol consumption (T0: 34.8%, n = 125; T1: 9.7%, n = 49), and psychoactive substance use (T0: 36.8%, n = 132; T1: 13.8%, n = 85.3).

## Discussion

Out results reveal notable shifts within the RAPS of Itatiba, particularly concerning the established pathways between various healthcare services, the operational procedures within certain services, and the provision of clinical care to users. These results align with the expectations of a matrix support implementation which by promoting interaction between services at different levels of care, especially those specialized in mental health and primary care, expands the possibilities for coordination between services and promotes comprehensive care ^17^ ^,^ ^18^.

Although the sociodemographic profile of the population served remained relatively stable between T0 and T1, there was a significant decrease in the number of patients treated in the outpatient clinic. Considering the premises involved in matrix support execution, this reduction may be related to the reconfiguration of the mental health network and a tendency towards less dependence on the outpatient clinic, which manages mild and moderate cases ^19^. Such a change suggests a greater absorption of mental health cases, especially milder ones, by primary care which would relieve the outpatient clinic’s burden. However, we cannot confirm this hypothesis as our study did not evaluate whether there was an actual increase in mental health care in primary care in the studied municipality.

Nevertheless, our results reveal significant changes in the source of patient referrals to each service, indicating a restructuring of the mental health network in Itatiba. Reduction in spontaneous admissions to CAPS II and the outpatient clinic suggests a reconfiguration in how patients access treatment. This change may be related to improved coordination mechanisms between different levels of mental health care, promoting a flow more aligned with the expectations of a tiered and organized health system like the Brazilian Unified National Health System (SUS, acronym in Portuguese) ^20^ ^,^ ^21^.

This perspective aligns with the findings of a recent integrative review on the effect of collaborative care models in mental health on outcomes observed among primary care professionals ^22^. According to the study, the implementation of collaborative care proposals increased formal and informal relations among different team members, thus enhancing communication and possibilities for case discussions.

Notably, the interaction between specialized services themselves also emerged as a relevant point in our study, with a decrease in patients coming from other specialized services to CAPS II, while CAPS AD and the outpatient clinic showed an increase in patients admitted through this type of referral. This finding supports the hypothesis of a shift in the studied health network from a centralized operation in the outpatient clinic, which previously functioned as a gatekeeper responsible for referring patients to other specialized services, especially CAPS II.

Additionally, it is important to highlight the decrease in the participation of private services, strongly represented by therapeutic communities, in referring patients to CAPS AD. This is a particularly notable change given that the relation between the health network and therapeutic communities has been one of the main contentious points in recent discussions about mental health policy in Brazil ^23^. Often linked to religious denominations, therapeutic communities are institutions dedicated to treating people with disorders related to alcoholism and other drug use which have faced criticism due to controversial practices and poor conditions, including reports of human rights violations, abuse, and non-scientific treatments ^23^ ^,^ ^24^. The lack of adequate regulation and supervision contributes to these problems, putting patients’ integrity at risk ^24^.

In this context, referrals to public health services generally do not represent an integration effort but rather the use of the public system to compensate for the lack of technical teams in these services concerning the validation of admissions by a health team. An aspect that aligns with this perspective is the type of therapeutic offerings received by CAPS AD patients in T0, strongly concentrated in group attendances, with a low proportion of patients receiving mental health medical consultations. In this sense, the changes occurring from the matrix support implementation denote not only an important articulation of the health network but also a significant review of the internal work processes of mental health services.

The changes in therapeutic offerings received by CAPS AD users indicate a significant transformation in how services are provided. The significant increase in the proportion of patients receiving mental health medical consultations and individual attendances with multidisciplinary team professionals reflects a shift towards a more patient-centered personalized care. But this change is accompanied by a decrease in group attendances which may negatively impact the availability of group treatments, recognized as a valuable therapeutic approach in various mental health contexts ^25^ ^,^ ^26^. Notably, the offer of group attendances in CAPS II remained low in both evaluated periods which may be partly related to the lack of appropriate physical space. During the study period, CAPS II operated in a former primary care unit, which did not meet the recommendations for the physical configuration of a CAPS, lacking adequate spaces for group activities ^27^.

Our results point to a significant increase in the number of patients receiving home visits in both CAPS AD and CAPS II between T0 and T1. This suggests greater adoption of care approaches based on the psychosocial paradigm, which values resources outside the services and a territorial perspective. Home visits are highlighted in the literature as a practice that promotes the reorganization of work processes and expands the possibilities for team action ^28^. This approach facilitates creating partnerships and strengthens intersectoral articulation, promoting a more integrated and comprehensive care network. By facilitating a solid structure for collaboration between teams from different services, the matrix support may have driven these activities.

Moreover, it is noted an increase in clinical care provided to patients at CAPS II and the outpatient clinic. The physical health of patients with mental disorders has been a global concern since mental health services tend to focus mainly on treating psychiatric conditions, neglecting crucial physical health issues like chronic diseases, lifestyle habits, and monitoring vital signs ^29^. This gap can compromise the overall quality of care, especially considering that patients with mental disorders have a higher prevalence of physical comorbidities and are less likely to access physical health services ^7^. Contributing to the mortality gap, this disparity reinforces the need to integrate mental and physical health services to offer more comprehensive care ^29^ ^,^ ^30^. In this sense, we believe that matrix support implementation played a key role in achieving these results, promoting this perspective of integration to enhance care and include this type of discussion in treatment plans.

Notably, this reality was not repeated in CAPS AD, where the provision of clinical care and the recording of physical comorbidities decreased. On the other hand, there was a significant improvement in CAPS AD records concerning smoking, alcoholism, and the use of psychoactive substances, with a significant decrease in the proportion of patients without records in these areas. This progress, coupled with an increase in diagnoses related to the abuse of psychoactive substances, points to greater diagnostic accuracy and a more focused approach to this population’s specific issues. However, it also reveals a greater need for efforts to ensure that physical comorbidity care and monitoring are not neglected.

While some areas showed less expressive advances in CAPS AD, we highlight, as in CAPS II, substantial changes in the mode of operation and articulation with the service network. As evidenced in the literature on previous experiences with the implementation of collaborative care proposals like matrix support ^22^ ^,^ ^31^, we observed improvements in the internal organization of services, with a reconfiguration of professional practices and systemic benefits, promoting greater access and exchange of information that can optimize the outcomes achieved with patients.

Some limitations must be considered when interpreting our results. Firstly, the analysis was based on data extracted from medical records which may result in biases due to a lack of standardization and detail in documentation. Additionally, this research was designed as a repeated cross-sectional study with two measurement points, which implies limitations related to causality and temporality. Although this type of study can give insights into observed changes, it does not necessarily establish a direct causal relation between the intervention and the outcomes, as uncontrolled or unforeseen variables may have influenced the results between the evaluated periods. Finally, the lack of information on possible changes in the provision of mental health care in primary care and the absence of specific information on the involvement of workers from each service in matrix support hindered understanding the penetration level of this arrangement in the studied context.

But this study presents important strengths, particularly its capacity to capture and analyze concrete changes in the configuration and functioning of a local mental health network over time, based on real-world service data. By employing a repeated cross-sectional design and working with multiple data sources across specialized services, the study provides a comprehensive overview of service articulation, care practices, and patient profiles in the context of matrix support implementation. These findings contribute to the growing body of knowledge on the organization of psychosocial care networks and may inform evaluations and planning in other municipalities or health systems seeking to strengthen mental health integration within primary care. The methodological approach adopted here can also serve as a model for similar studies seeking to assess service restructuring and interprofessional collaboration in mental health care.

## Conclusions

Implementation of a matrix support in the studied municipality has shown significant effects on RAPS functioning, with improvements in various aspects of care delivery. Results indicate substantial changes in the origin of referrals, with greater involvement of primary care and integration among specialized services contributing to a more coordinated and effective care approach. Additionally, we observed a significant improvement in the clinical care provided to patients, with increased attention to vital signs measurement, anamnesis, and physical examination, as well as improved records of smoking, alcohol consumption, and psychoactive substance use.

Our findings suggest that implementing a matrix support can contribute to the improvement, organization, and effectiveness of mental health services, promoting greater access and quality of care for users. In this sense, the results underscore the importance of health policies that encourage collaborative and integrated practices within the mental health network, intended to promote approaches that contribute to a more comprehensive care experience for individuals with mental disorders.
